# Assessment of hydrochemical characteristics, health risks and quality of groundwater for drinking and irrigation purposes in a mountainous region of Pakistan

**DOI:** 10.1007/s11356-024-34046-7

**Published:** 2024-06-25

**Authors:** Waqar Azeem Jadoon, Muhammad Zaheer, Abdul Tariq, Raja Umer Sajjad, Memet Varol

**Affiliations:** 1 Department of Earth & Environmental Sciences, Hazara University, Mansehra, 21120 Khyber Pakhtunkhwa Pakistan; 2 Key Laboratory of Mechanics On Disaster and Environment in Western China, the Ministry of Education of China, Lanzhou University, Lanzhou, 730000 China; 3 Department of Mechanics, College of Civil Engineering and Mechanics, Lanzhou University, Lanzhou, 730000 China; 4 Engineering and Management Sciences, Balochistan University of Information Technology, Quetta, 87300 Balochistan Pakistan; 5 Agriculture Faculty, Aquaculture Department, Malatya Turgut Özal University, Malatya, Türkiye

**Keywords:** Groundwater, Health risk assessment, Hydrochemistry, Hydrological facies, Irrigation water, Water quality indices

## Abstract

Renowned for its agriculture, livestock, and mining, Zhob district, Pakistan, faces the urgent problem of declining groundwater quality due to natural and human-induced factors. This deterioration poses significant challenges for residents who rely on groundwater for drinking, domestic, and irrigation purposes. Therefore, this novel study aimed to carry out a comprehensive assessment of groundwater quality in Zhob district, considering various aspects such as hydrochemical characteristics, human health risks, and suitability for drinking and irrigation purposes. While previous studies may have focused on one or a few of these aspects, this study integrates multiple analyses to provide a holistic understanding of the groundwater quality situation in the region. Additionally, the study applies a range of common hydrochemical analysis methods (acid–base titration, flame atomic absorption spectrometry, and ion chromatography), drinking water quality index (WQI), irrigation indices, and health risk assessment models, using 19 water quality parameters. This multi-method approach enhances the robustness and accuracy of the assessment, providing valuable insights for decision-makers and stakeholders. The results revealed that means of the majority of water quality parameters, such as pH (7.64), electrical conductivity (830.13 μScm^–1^), total dissolved solids (562.83 mgL^–1^), as well as various anions, and cations, were in line with drinking water norms. However, the water quality index (WQI) predominantly indicated poor drinking water quality (range = 51–75) at 50% sites, followed by good quality (range = 26–50) at 37% of the sites, with 10% of the sites exhibiting very poor quality (range = 76–100). For irrigation purposes, indices such as sodium percent (mean = 31.37%), sodium adsorption ratio (mean = 0.98 meqL^–1^), residual sodium carbonate (– 3.15 meqL^–1^), Kelley’s index (mean = 0.49), and permeability (mean = 49.11%) indicated suitability without immediate treatment. However, the magnesium hazard (mean = 46.11%) and potential salinity (mean = 3.93) demonstrated that prolonged application of groundwater for irrigation needs soil management to avoid soil compaction and salinity. Water samples exhibit characteristics of medium salinity and low alkalinity (C2S1) as well as high salinity and low alkalinity (C3S1) categories. The Gibbs diagram results revealed that rock weathering, including silicate weathering and cation exchange, is the primary factor governing the hydrochemistry of groundwater. The hydrochemical composition is dominated by mixed Ca–Mg–Cl, followed by Na–Cl and Mg–Cl types. Furthermore, the human health risk assessment highlighted that fluoride (F^–^) posed a higher risk compared with nitrate (NO_3_^–^). Additionally, ingestion was found to pose a higher risk to health compared to dermal contact, with children being particularly vulnerable. The average hazard index (HI) for children was 1.24, surpassing the allowable limit of 1, indicating detrimental health effects on this subpopulation. Conversely, average HI values for adult females (0.59) and adult males (0.44) were within safe levels, suggesting minimal concerns for these demographic groups. Overall, the study’s interdisciplinary approach and depth of analysis make a significant contribution to understanding groundwater quality dynamics and associated risks in Zhob district, potentially informing future management and mitigation strategies.

**Supplementary Information:**

The online version contains supplementary material available at 10.1007/s11356-024-34046-7.

## Introduction

Access to safe drinking water has gained global attention in recent decades, a concern particularly critical in water-scarce Pakistan. A staggering 1 billion people around the world lack access to safe drinking water, intensifying the challenge of ensuring their well-being (Yang et al. 2023). Significantly, groundwater emerges as a lifeline for over 1.5 billion individuals and supports more than 40% of irrigation needs globally (Zhao et al. 2022). Especially in arid regions where surface water is scarce, groundwater stands as the primary source for drinking, domestic, and irrigation use (Khan et al. 2023). Over-extraction and contamination, stemming from both natural processes and human activities, deteriorate groundwater quality and contribute to its depletion. These issues not only jeopardize ecological balance and human well-being but also cast limitations on its use (Salem et al. 2023; Yang et al. 2023). Therefore, to leave clean and sufficient water to future generations, much attention has been paid to its protection and rational use (Çankaya et al. 2023; Uddin et al. 2023).

Groundwater availability relies on quantity as well as its hydrochemical makeup. Geological and hydrological processes together shape groundwater quality (Sinha et al. 2023; Zhao et al. 2022). Elevated chemical levels in groundwater can gravely compromise its quality (Jafarzadeh et al. 2022). Groundwater containing excessive fluoride, chlorides, bicarbonates, nitrates, sodium, calcium, magnesium, and heavy metals poses health risks and threatens soil fertility and crop yields (Rushdi et al. 2023; Yang et al. 2023). Prior studies analyze natural and human-induced impacts on water quality via statistical and hydrogeochemical modeling (Farid et al. 2022; Kumar et al. 2020; Lanjwani et al. 2022; Salem et al. 2021; Samtio et al. 2022; Sinha et al. 2023; Uddin et al. 2023). A comprehensive water quality assessment facilitates efficient water resource management, thereby conserving valuable financial resources that would otherwise be allocated to water treatment. Additionally, it contributes to improved crop quality and yield (Omeka 2023). The presence of contaminated water poses a significant challenge to ensuring safe and sufficient drinking, irrigation, and industrial water supplies worldwide, affecting policymakers and end-users alike. Hence, it is imperative to urgently address these issues through rapid and cost-effective monitoring, along with the implementation of robust integrated methods for effective management, thereby ensuring the sustainability of available water resources (Omeka 2023). Thus, assessing water quality becomes vital for human health, sustainable agriculture, and sustainable economic growth (Çankaya et al. 2023).

Classically, water quality assessment is based on the comparison of the values of hydrochemical parameters obtained as a result of the analysis of water with national and international water quality criteria (Varol & Tokatlı, 2023). Parameters like color, odor, EC, pH, turbidity, alkalinity, potassium (K^+^), bicarbonate (HCO_3_^–^), calcium (Ca_2_^+^), nitrate (NO_3_^–^), carbonate (CO_3_^–^), chloride (Cl^–^), magnesium (Mg^2+^), hardness, sodium (Na^+^), total dissolve solids (TDS), sulfate (SO_4_^2–^), and fluoride (F^–^) are crucial in hydrochemical analysis. Notably, Na^+^, K^+^, NO_3_^−^, and F^−^ concentrations are significant groundwater contaminants (Samtio et al. 2022; Zhao et al. 2022). For assessing groundwater suitability for irrigation, parameters, like soil adsorption ratio (SAR), sodium percentage (Na%), residual sodium carbonate (RSC), Kelly’s index (KI), TDS, total hardness, permeability index (PI), magnesium hazard (MH), and potential salinity (PS), are widely utilized (Panneerselvam et al. 2021; Unigwe et al. 2022).

The application of individual parameters may not provide a comprehensive assessment of water quality, as one parameter’s adequacy could mask deficiencies in another (Çankaya et al. 2023). Consequently, depending exclusively on conventional methods for assessing water quality may not yield the precise and effective outcomes required for accurately predicting water quality and ensuring resource sustainability (Omeka 2023). To address this issue, the water quality index (WQI) comes into play, offering a holistic evaluation by considering multiple physicochemical parameters (Haq & Muhammad 2023; Ram et al. 2021; Shaibur et al. 2024). WQI serves as an effective tool, simplifying intricate water quality data into a comprehensible single value for policymakers and environmentalists (Jehan et al. 2023). Operating on a scale of 0 to 100, the WQI encapsulates complex information, concisely rating water quality status (excellent, good, poor, etc.) at specific locations and times (Uddin et al. 2023). The idea is based on the notion that a lower value indicates a smaller deviation from the suggested parameter values, resulting in higher quality water suitable for human consumption and vice versa (Shaibur et al. 2024). WQI reflects the composite effect of multiple water quality parameters on the overall water quality (Kumar et al. 2020; Shahab et al. 2018). The WQI is very much interrelated with human health. Humans can be exposed to pollutants found in groundwater through drinking and the dermal contact with this water. In this respect, the evaluation of groundwater in terms of health risk is of great importance for the protection of public health (Varol & Tokatlı, 2023).

Contaminated groundwater represents a significant hazard, particularly in developing nations, contributing to various waterborne illnesses (Lanjwani et al. 2022). In Pakistan, a staggering 50% of diseases and 40% of deaths are associated with the consumption of subpar water, affecting one in every five individuals (Lanjwani et al. 2022). Assessing the human health risks linked to groundwater contamination is imperative, offering a vital link between environmental pollution and public health outcomes (Igwe & Omeka 2022; Omeka & Egbueri 2023). This assessment is essential for environmental health management, particularly in regions where water sources are compromised. It involves evaluating potential health hazards posed by groundwater contaminants and assessing the probability of adverse health effects in exposed populations. Various tools such as the human health risk assessment (HHRA) model, average daily intake (ADI), hazard quotients (HQs), and hazard index (HI) play a vital role in this process, providing invaluable insights into potential health risks (Adebayo et al. 2021; Jehan et al. 2023; Tomašek et al. 2022). By identifying vulnerable populations and prioritizing mitigation measures, HHRA plays a central role in safeguarding public health and guiding policy interventions to ensure safe and sustainable access to clean water resources.

Recent global attention has focused on groundwater contamination and associated health risks covering various countries including Bangladesh (Islam et al. 2018; Shaibur et al. 2024), India (Arumugam et al. 2023; Khan et al. 2023; Saraswat et al. 2023; Selvam et al. 2023), China (Lan et al. 2023), Nigeria (Igwe & Omeka 2022; Omeka 2023), and with a significant focus on Pakistan. Studies have highlighted groundwater contamination issues in various regions of Pakistan, including Balochistan, Larkana, Lahore, Peshawar, Karak, Muzaffarabad, and Gilgit-Baltistan (Ahmad et al. 2021; Hayder et al. 2022; Ismail et al. 2023; Javed et al. 2019; Khanoranga & Khalid 2019; Lanjwani et al. 2022; Sohail et al. 2019). However, to our best knowledge, the current study area lacks such investigations, which is critical to address, particularly in this water-scarce region.

Recently, geographical information system (GIS) emerged as a sophisticated and robust tool for illustrating water quality mapping and assessment. Previous studies have effectively harnessed GIS to analyze groundwater quality across diverse conditions and geographical regions (Arumugam et al. 2023; Ram et al. 2021). GIS offers an economically viable and time-efficient approach to transform extensive datasets into spatial distribution maps, revealing trends, associations, and sources of pollutants. Its role is crucial in both groundwater pollution risk assessment and groundwater quality planning (Ram et al. 2021). In our study, kriging geospatial interpolation method was employed to generate the spatial distribution maps of various groundwater parameters, WQI, and health risk.

Zhob district, located in the northwest of Balochistan (Pakistan), is an important region in terms of agriculture and animal husbandry. Since most of the region is mountainous, groundwater is used for agricultural irrigation (Ashraf et al. 2021). For this reason, groundwater is a vital resource for drinking and irrigation in the district. The significance of this study lies in its comprehensive assessment of hydrochemical characteristics, health risks, and groundwater quality for both drinking and irrigation purposes in this mountainous region. Addressing the critical need for understanding groundwater dynamics in regions with complex geological and hydrological settings, this study is not only pertinent to local communities but also to the broader international scientific community. It contributes to the global body of knowledge on groundwater dynamics, particularly in mountainous terrains, which are often underrepresented in scientific literature and water resource management. Moreover, it provides vital information for public health interventions, aligning with and contributing to the achievement of Goal 6 (Clean Water and Sanitation) and Goal 3 (Good Health and Well-being) of the Sustainable Development Goals (SDGs). Additionally, this study can contribute to transboundary water management efforts, promoting collaboration and cooperation among neighboring countries. This study brings a novel perspective by integrating considerations of human health risk, alongside spatial hydrochemistry analysis in the Zhob district—previously unreported. Moreover, the research assesses groundwater quality for its applicability across diverse sectors and evaluates the associated risks stemming from contaminated water usage. The overall aim of this study is to conduct a comprehensive assessment of hydrochemical characteristics, spatial variability in water quality using GIS, and groundwater suitability for domestic and agricultural purposes through WQI and irrigation indices and to investigate the non-cancer health risks associated with the consumption of NO_3_^–^ and F^–^ contaminated water. The study’s specific objectives include the following: (i) to gain a comprehensive insight into the hydrochemistry and groundwater facies; (ii) to carry out a spatial variability of water quality in the study area using GIS-based spatial models; (iii) to evaluate groundwater’s fitness for domestic and agricultural uses through WQI and irrigation indices; and (iv) to investigate the non-cancer health risks associated with the consumption of NO_3_^–^ and F^–^ contaminated water. The outcomes of this research will enhance water quality evaluation in the surrounding region, offering valuable input for both immediate and long-term planning aimed at water quality protection, management, sustainability, and safeguarding public health. focusing water quality protection, management, sustainability, and public health protection.

## Material and methods

### Study area

District Zhob is situated (30° 26′ 54″–31° 57′ 8″ N and 67° 48′ 41″– 69° 44′ 43″ E) about 430 km (aerial distance) southwest of Islamabad at an elevation of 1408 m above mean sea level (MSL) in northwest Balochistan. The district features a train composed of mountains and valleys spanning an elevation range of 930 to 2658 m above MSL. The total area of district Zhob is 15,987 km^2^ with a population of 310,544 (Ashraf et al. 2021). Divided into two major subdivisions—Tehsil Kakar Khurasan and Tehsil Zhob—the district encompasses an agricultural area of 126,719 hectares (6.23% of geographical area), while most of the area is barren land and mountainous (Ashraf et al. 2021). Key crops include wheat, rapeseed/mustard, barley, vegetables, fodder, sorghum, maize, pulses, melons, and chilies. The region also boasts fruits such as apples, grapes, almonds, and apricots. Livelihoods primarily hinge on agriculture, livestock, and mining, all of which are heavily reliant on groundwater. Groundwater provides approximately 90% of the drinking water. Characterized by a hyper-arid to semi-arid climate, the region experiences dry, cold winters, and scant annual rainfall of about 285 mm, primarily occurring in July and August (Ashraf et al. 2021).

### Geology and hydrology of the study area

**Fig. 1:**
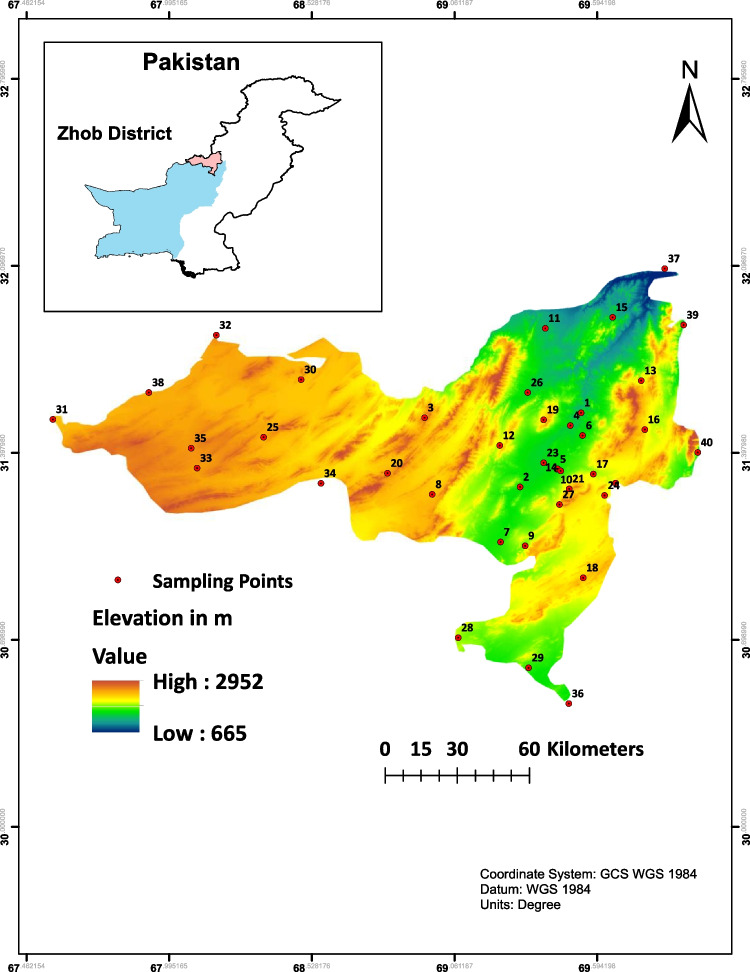
Study area map

Geologically, the study area constitutes the northern segment of the Suleiman fold belt (SFB), situated at the western margin of the Indian plate in Pakistan. The SFB is a broad (> 300 km) fold and thrust belt that originated in a transgressional environment. The rocks in Zhob Valley are dominantly composed of intensely fractured mafic–ultramafic rocks hosting chromite, sulfide mineralization, and metamorphic rocks (Ullah et al. 2019). Inherent to Zhob are mineral concessions for chromite, coal, and granite. Additionally, reports indicate the presence of glass sand, copper, feldspar, calcite, fluorite, phosphate rocks, limestone, soapstone, manganese, and laterite. Within these fractured rocks (mafic–ultramafic and clastic sedimentary rocks (shale, sandstone, and siltstone)), aquifer emerges as a consequence of weathering, alteration, and dissolution, becoming a dynamic contributor to groundwater in the region (Khan et al. 2010). The map of the study area is provided in Fig. 1. The Zhob River, coursing through the district, stretches approximately 410 km in length. Originating from the Kan Metarzai Range, the river ultimately merges into the Gomal River.

### Groundwater sampling and analysis

In total, 40 composite groundwater samples (*n* = 3) were collected in 1.5L HDP bottles from distinct locations across district Zhob. Before sampling, the water was run for 10 min to purge any potential foreign contaminants and establish a steady state, ensuring that the obtained sample accurately represents the water quality (Omeka & Egbueri 2023; Shaibur et al. 2024). The collection bottles were immersed in 10% nitric acid for 24 h, followed by thorough washing using distilled water. Furthermore, the bottles were rinsed with the source water to be sampled prior to collection, ensuring full acclimatization of the bottle conditions with the sampling sites (Omeka & Egbueri 2023; Shaibur et al. 2024). The tightly sealed and properly labeled sampled bottles were transported to the laboratory under controlled temperature (below 4 °C) for ions analysis, following established protocols (APHA 2017). The assessment of water quality encompassed an examination of numerous parameters such as water color, odor, taste, turbidity, pH, EC, TDS, and concentrations of ions including Na^+^, K^+^, Ca^2+^, Mg^2+^, SO_4_^2–^, HCO_3_^–^, NO_3_^–^, CO_3_^2–^, Cl^–^, F^–^, and hardness. Certain parameters like color, odor, taste, turbidity, pH, EC, and TDS were promptly measured at the sampling sites, while others were analyzed in the laboratory. The cations and anions were analyzed using flame atomic absorption spectrometry, acid–base titration, and ion chromatography following previously established protocols (APHA 2017; Yang et al. 2023), respectively.

### Quality assurance and control

Quality assurance and control measures were rigorously enforced, including the utilization of deionized water, calibrated laboratory equipment, clean labwares, and chemicals of analytical grade. Each batch, consisting of 10 samples, underwent correction based on the average procedural blank concentrations (distilled water) (*n* = 3) processed alongside the corresponding real sample batch. Calibration standards were run after every 10th sample to ensure instrument sensitivity and stability. Throughout the experiment, analyte signal intensities remained consistently reliable, with a relative standard deviation (RSD) of less than 5%, demonstrating instrument sensitivity and stability. Furthermore, the analytical precision of each groundwater sample was validated through the assessment of charge balance error (%CBE = (∑cations – ∑anions / ∑cations + ∑anions) × 100). The resulting CBE percentages were within the suitable range of ± 10 (Omeka & Egbueri 2023).

### Groundwater quality for drinking

Groundwater quality was computed to evaluate its suitability for drinking purposes. Initially, weights (wi) were assigned to various parameters on a scale of 1–5, considering the severity of their impact on human health (Table 1) (Zhao et al. 2022). Subsequently, the relative weights (Wi) are computed as follows:

**Table 1:** Descriptive statistics of physiochemical parameters, their quidelines and the parameter for water quality index (WQI) calculation assessment in the groundwater of the District Zhob

Parameter	Min	Max	Mean	SD	CV%	Skewness	Kurtosis	WQI		WHO (2022)	USEPA (2017)
								w_i_	W_i_		
pH	6.9	8.4	7.64	0.28	3.82	0.03	2.48	3.7	0.09	6.5–8.5	6.5–8.5
EC (μScm^–1^)	390	1703	830.13	368.37	44.38	0.99	0.39	3.2	0.07	1000	500
TDS (mgL^–1^)	232	1719	562.83	322.94	57.38	1.89	4.54	4.3	0.10	1000	
Na^+^ (mgL^–1^)	3.0	190	61.87	38.79	62.7	1.53	3.36	2.9	0.07	200*	200
K^+^ (mgL^–1^)	0.0	6.0	3.57	2.14	60.13	-0.10	-1.63	2.0	0.05	12	
Mg^2+^ (mgL^–1^)	2.0	120	34.89	25.2	72.22	1.59	3.42	2.3	0.05	50	
Ca^2+^ (mgL^–1^)	28	178	65	39.06	60.09	1.73	2.89	2.4	0.06	75	
F^–^ (mgL^–1^)	0.0	2.0	0.55	0.42	77.33	1.64	2.88	5.0	0.12	1.5	
Cl^–^ (mgL^–1^)	10	650	81.43	115.02	141.25	4.32	22.81	3.5	0.08	250*	250
SO_4_^2–^ (mgL^–1^)	48	426	156.67	94.96	60.61	1.35	2.43	3.9	0.09	250**	250
HCO_3_^–^ (mgL^–1^)	100	310	183.33	46.93	25.60	0.64	1.08	2.2	0.05	500	
CO_3_^2–^ (mgL^–1^)	0.0	5.6	0.19	1.02	547.72	5.30	31.00				
NO_3_^–^ (mgL^–1^)	0.0	9.7	2.61	2.24	424.32	1.54	3.52	4.9	0.11	45	
Hardness (mgL^–1^)	58	500	285.8	125.02	43.74	0.49	-0.87			500	
Turbidity (NTU)	1.8	15.8	7.99	3.05	38.22	0.91	1.94	2.5	0.06	5	

wi is the assigned of the parameters based on their impact on human health and the purpose of drinking water

Wi is the relative weight

^*^No health-based guideline is proposed, but a taste threshold exists

^**^No health-based guideline is proposed for SO_4_^2–^; the threshold is for taste, corrosion, and gastrointestinal effects resulted from higher SO_4_^2–^ levels

1 $${\mathrm{W}}_{\mathrm{i}}=\frac{{\mathrm{w}}_{\mathrm{i}}}{{\sum }_{\mathrm{i}=1}^{\mathrm{n}}{\mathrm{w}}_{\mathrm{i}}}$$

W_i_ represents the relative weight, w_i_ is the assigned weight of an individual parameter, and *n* denotes the number of parameters.

Thereafter, the quality rating scale (qi) of each water quality parameter is determined as follows:

2 $${\mathrm{q}}_{\mathrm{i}}=\left(\frac{{\mathrm{C}}_{\mathrm{i}}}{{\mathrm{S}}_{\mathrm{i}}}\right)\mathrm{x }100$$

where Ci is the observed concentration (mgL^–1^) of each parameter and Si is their corresponding WHO standard limit.

Finally, the subindex (SI) is calculated to get the water quality index (WQI) as follows:

3 $$\mathrm{SI}={\mathrm{W}}_{\mathrm{i}}\mathrm{ x }{\mathrm{q}}_{\mathrm{i}}$$

4 $$\mathrm{WQI}=\sum {\mathrm{SI}}_{\mathrm{i}}$$

### Groundwater quality for irrigation

The Zohb district holds significant agricultural importance, requiring a specific volume of water for effective irrigation. However, both the quantity and quality of water significantly influence soil sustainability and crop growth and yield. The utilization of unsuitable groundwater for irrigation could lead to soil degradation and reduced crop yield, making it crucial to utilize high-quality irrigation water (Panneerselvam et al. 2021). Thus, evaluating the suitability of groundwater for irrigation becomes imperative for ensuring sustainable agricultural development. This involves the calculation of Na%, SAR, RSC, KI, PI, MH, and PS, as outlined below:

5 $$\mathrm{Na\%}=\frac{{\mathrm{Na}}^{+}+ {\mathrm{K}}^{+}}{{\mathrm{Ca}}^{2+} +\mathrm{ M}{\mathrm{g}}^{2+} +\mathrm{ N}{\mathrm{a}}^{+ }+ {\mathrm{K}}^{+}}\mathrm{x }100$$

6 $$\mathrm{SAR}=\frac{{\mathrm{Na}}^{+}}{\sqrt{\frac{{\mathrm{Ca}}^{2+}+{\mathrm{Mg}}^{2+}}{2}}}$$

7 $$\mathrm{RSC}=\left({\mathrm{HCO}}_{3}^{-} +{\mathrm{CO}}_{3}^{2-}\right)-\left({\mathrm{Ca}}^{2+}+{\mathrm{Mg}}^{2+}\right)$$

8 $$\mathrm{KI}=\frac{\left({\mathrm{Na}}^{+}\right)}{\left({\mathrm{Ca}}^{2+} +\mathrm{ M}{\mathrm{g}}^{2+}\right)}$$

9 $$\mathrm{PI}=\frac{{\mathrm{Na}}^{+}+\sqrt{{\mathrm{HCO}}_{3}^{-}}}{{\mathrm{Ca}}^{2+}+\mathrm{ M}{\mathrm{g}}^{2+}+{\mathrm{Na}}^{+}}\mathrm{ x }100$$

10 $$\mathrm{MH}=\frac{{\mathrm{Mg}}^{2+}}{({\mathrm{Ca}}^{2+}+\mathrm{ M}{\mathrm{g}}^{2+})}\mathrm{ x }100$$

11 $$\mathrm{PS}={\mathrm{Cl}}^{-}+0.5\mathrm{ x S}{\mathrm{O}}_{4}^{2-}$$

Detailed information of these indices is provided in Supplementary Text S1, and their classification is given in Table 2. Furthermore, Wilcox and United States Salinity Laboratory (USSL) diagrams were generated to evaluate the suitability of the groundwater for irrigation.

**Table 2:** Classification of drinking and irrigation water quality evaluation indices and their results for the groundwater of the study area

Parameter	Range	Category	Min	Max	Mean	Percent of samples
Drinking water quality						
DWQI	0–25	Excellent	38.02	101.88	57.75	0
	26–50	Good				36.67
	51–75	Poor				50
	76–100	Very poor				10
	> 100	Undrinkable				3.33
Irrigation water quality						
Sodium percentage (Na%)	0–20%	Excellent	0.72	60.34	31.37	6.67
	20–40%	Good				76.67
	40–60%	Permissible				16.67
	60–80%	Doubtful				0
	> 80%	Unsuitable				0
Sodium adsorption ratio (SAR)	0–10	Excellent	0.01	2.98	0.98	100
	10–18	Good				0
	18–26	Doubtful				0
	> 26	Unsuitable				0
Residual sodium carbonate (RSC)	< 1.25	Good	–13.12	–0.79	–3.15	100
	1.25–2.5	Doubtful				0
	> 2.5	Unsuitable				0
Kelley’s index	< 1	Suitable	0.01	1.49	0.49	93
	> 1	Unsuitable				7
Permeability index (PI)	> 75%	Excellent (class I)	13.45	74.0	49.11	0
	25–75%	Suitable (class II)				97
	< 25%	Unsuitable (class III)				3
Magnesium hazard (MH)	< 50%	Suitable	4.10	81.70	46.11	53.33
	> 50%	Unsuitable				46.67
Potential salinity (PS)	< 3	Suitable	1.18	19.34	3.93	46.67
	> 3	Unsuitable				53.33

### Hydrochemical facies analysis

For the analysis and interpretation of water quality, the Aqua-Chem (11.0) software was employed, making it a pertinent tool for assessing hydrochemical facies. Through Aqua-Chem, a Piper plot was generated, offering a graphical framework to classify various water types. The Piper plot facilitates the categorization of groundwater samples into six distinct facies based on their chemical composition. The arrangement of samples within the plot revealed a predominant concentration in a particular zone, thereby aiding the identification and evaluation of water quality.

### Human health risk assessment (HHRA)

The non-cancer risk of exposure to groundwater pollutants through ingestion and dermal contact for residents in the study area was estimated using the HHRA model (Qu et al. 2022; Selvam et al. 2023). This model is often preferred for several reasons. Firstly, it provides a comprehensive framework for evaluating potential health risks associated with exposure to various contaminants in different environmental media such as air, water, soil, and food. Secondly, HHRA considers multiple pathways of exposure, including ingestion, inhalation, and dermal contact, which allows for a more thorough assessment of risk. Additionally, HHRA incorporates factors such as exposure duration, frequency, and concentration levels to estimate the likelihood and severity of health effects. Moreover, the HHRA model is adaptable and can be tailored to specific scenarios and populations, making it a versatile tool for risk assessment in diverse contexts. Lastly, HHRA outputs are often presented in a format that is accessible to policymakers, stakeholders, and the general public, facilitating informed decision-making and risk management strategies. Overall, the HHRA model is preferred due to its comprehensive approach, flexibility, and utility in assessing and mitigating potential health risks associated with environmental exposures.

The HHRA model encompassed four key steps: hazards identification, dose–response analysis, exposure assessment, and risk characterization (Shaibur et al. 2024; Yang et al. 2022). The initial two steps involved the selection of NO_3_^–^ and F^–^ to evaluate their potential health risks for different resident groups, namely children, adult females, and adult males. These groups were categorized based on individual characteristics such as exposure frequency, duration, ingestion rate, and body weight (Qu et al. 2022; Yang et al. 2022).

The third step (exposure assessment) involved the determination of the average daily intake (ADI/mg kg^–1^ day^–1^) through both ingestion and dermal contact pathways. This is calculated using the formula:

12a $${\mathrm{ADI}}_{\mathrm{ingestion}}=\frac{\left({\mathrm{C}}_{w}\mathrm{ x }{\mathrm{IR}}_{\mathrm{ing}}\mathrm{ x EF x ED}\right)}{\mathrm{BW x AT}}$$

12b $${\mathrm{ADI}}_{\mathrm{dermal}}=\frac{\left({\mathrm{C}}_{\mathrm{w}}\mathrm{ x }{\mathrm{S}}_{\mathrm{a}}\mathrm{ x }{\mathrm{K}}_{\mathrm{p}}\mathrm{ x T x EV x EF x ED x CF}\right)}{\mathrm{BW x AT}}$$

where the explanation of parameters and their value used in calculations are given in Table S1.

The hazard quotient (HQ) for both ingestion and dermal intake is expressed as follows:

13a $${\mathrm{HQ}}_{\mathrm{Ingestion}}=\frac{{\mathrm{ADI}}_{\left(\mathrm{Ingestion}\right)}}{{\mathrm{RfD}}_{\mathrm{o}}}$$

13b $${\mathrm{HQ}}_{\mathrm{Dermal}}=\frac{{\mathrm{ADI}}_{(\mathrm{Dermal})}}{({\mathrm{RfD}}_{\mathrm{o }}\mathrm{x GIABS})}$$

where the explanation and values of RfDo for NO_3_^–^ and F^–^ and GIABS are given in Table S1.

In the concluding step of the HHRA model (step 4; risk characterization), the total hazard index (HI) is computed to assess the non-cancer risk, given by the following equation:

14 $$\mathrm{HI}=\sum {\mathrm{HQ}}_{\mathrm{ingestion}+\text{HQ dermal}}$$

### Statistical analysis

All statistical analyses, including assessments of data normality, basic statistics, and Pearson correlation analysis, were performed using R software (version 4.2.1; 2022).

## Results and discussion

### Hydrochemistry of groundwater

**Fig. 2:**
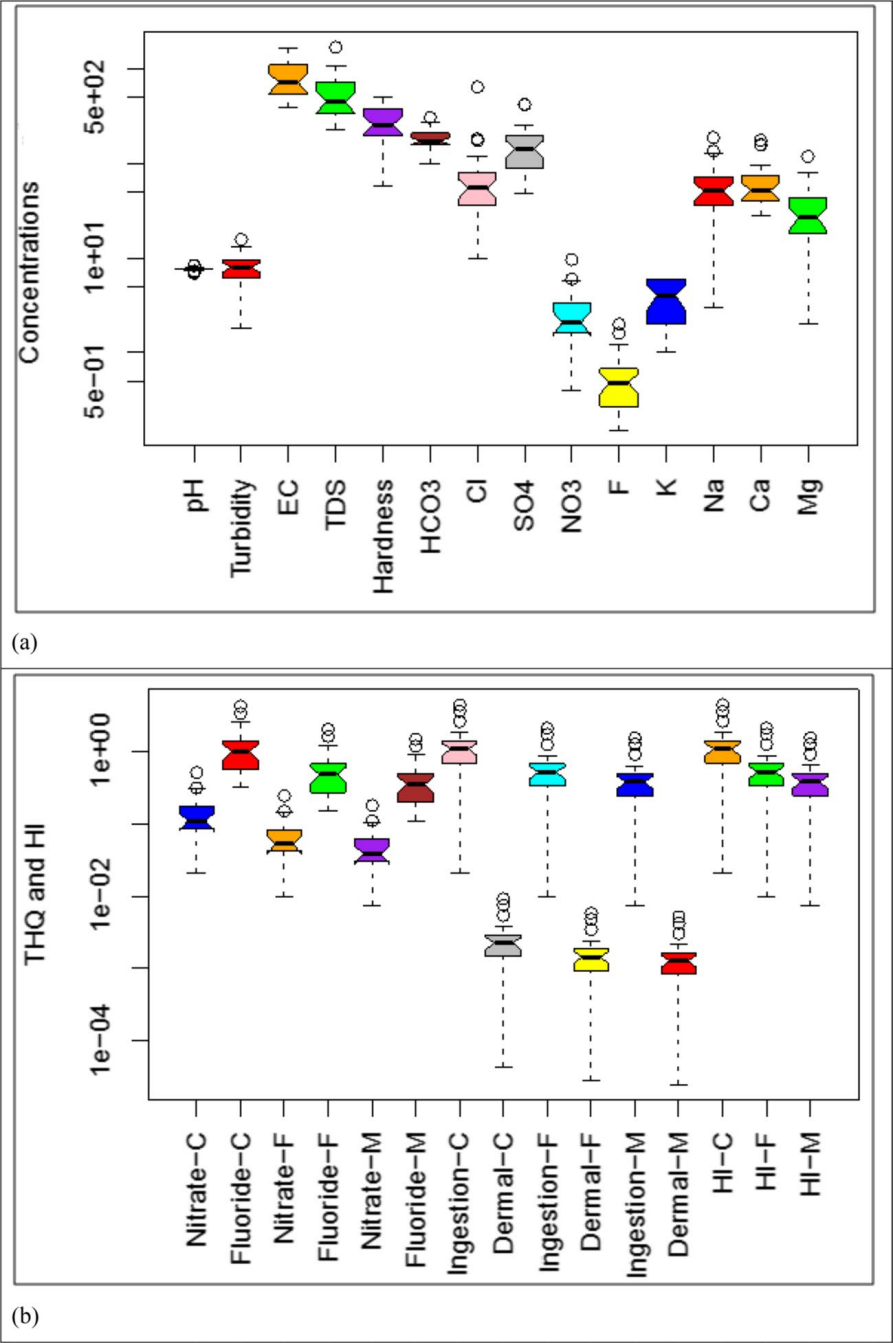
**a** Box plots of various groundwater quality parameters (units of all parameters: mg/L except pH, turbidity (NTU) and EC (µS/cm)) and **b** THQ calculated for chemical species (nitrates + fluorides), exposure pathways (ingestion + dermal contact), and hazard index for children (-C), adult females (-F) and adult males (-M)

The physicochemical parameters of groundwater hold significant importance in identifying its type, quality, and nature (Khanoranga & Khalid 2019). Analysis of these parameters revealed that water color was acceptable at 40% of the sites but problematic at 60%. However, both odor and taste were unobjectionable at all locations. The statistical summary of hydrochemical data and their recommended guidelines are presented in Table 1. More insights into pH, turbidity, EC, TDS, and hardness are provided in Fig. 2a. For pH, a vital parameter, the values ranged from 6.90 to 8.40, with an average of 7.64. All pH values met WHO’s guidelines (6.5–8.5) (WHO 2022). The water slightly leaned towards alkaline nature, potentially due to bicarbonates and calcium (Çankaya et al. 2023; Şener et al. 2017), aligning with past studies from Pakistan (Khanoranga & Khalid 2019; Samtio et al. 2022; Umar et al. 2013). However, Ghoraba and Khan (2013) reported a slightly higher pH (7.92) for Balochistan province. EC ranged from 390 to 1703 μScm^–1^, averaging 830.13 μScm^–1^. Similarly, TDS varied from 325 to 1719 mgL^–1^, averaging 562.83 mgL^–1^ (Fig. 2a). EC and TDS averages adhered to WHO's standards, but, individual EC value exceeded limits at 30% of sites and TDS exceeded at 10% (Table S2). When compared to USEPA (2017) standards, EC surpassed recommended levels at 93% of sites. High EC values demand attention, as they enhance groundwater’s mineral dissolution capacity, which can hinder plants’ ion absorption from the soil solution and lead to physiological drought (Naseem et al. 2010). Our EC and TDS findings align with previous studies (Kumar et al. 2019; Lanjwani et al. 2022), though our average EC is lower than the overall average of 1025.58 μScm^–1^ in Balochistan province (Ghoraba & Khan 2013). Hardness ranged from 58 to 500 mgL^–1^, averaging 285.8 mgL^–1^, within permissible limits. Our study’s hardness average is lower than Jacobabad’s 500 mgL^–1^ (Shahab et al. 2018). Turbidity, on average, was 7.99 NTU, and 83.33% of water samples had higher turbidity than the recommended 5 NTU for drinking water (WHO 2022).

Major anion concentrations, based on mean values (mgL^–1^), followed this order: HCO_3_^–^ (183.33) > SO_4_^2–^ (156.67) > Cl^–^ (81.43) > NO_3_^–^ (2.61) > F^–^ (0.55) > CO_3_^2–^ (0.19) (Fig. 2a), consistent with previous findings (Qu et al. 2022; Yang et al. 2023). HCO_3_^–^ remained within WHO’s limits at all sites. Nevertheless, Cl^–^ surpassed recommended levels at 3.33% of sites, F^–^ at 6.67%, and SO_4_^2–^ at 13.33% (Table S2). The presence of HCO_3_^–^ in groundwater suggests that carbonate dissolution predominantly shapes the groundwater’s chemical composition (Khanoranga & Khalid 2019). Carbon dioxide interacting with minerals in soil leads to bicarbonate, resulting in an alkaline groundwater environment (Ram et al. 2021). Chloride’s presence in groundwater is natural, but elevated levels signify contamination and human health hazards; hence, it acts as an important indicator of water quality. Sulfate ions dissolve into water from gypsum, iron sulfides, and sulfur-containing rocks, making their presence in groundwater a product of geological processes (Iqbal et al. 2023). Fluoride, a common component in groundwater, typically results from interactions between water and fluoride-containing rocks. The presence of granite rocks in the study area could contribute the fluoride in the groundwater. It is established that fluorite-bearing rocks account for fluoride ions (Khanoranga & Khalid 2019).

Analytical findings reveal that within the cation group, calcium dominates, followed by sodium in the sequence Ca^2+^ (39.32%) > Na^+^ (37.42%) > Mg^2+^ (21.11%) >  > K^+^ (1.16%) (Fig. 2a) (Jehan et al. 2023). Calcium and magnesium concentrations ranged from 28 to 178 mgL^–1^ and 2 to 120 mgL^–1^, respectively, with average levels of 65 mgL^–1^ and 34.89 mgL^–1^. These concentrations, primarily influenced by carbonate minerals, generally met permissible standards. Although Ca^2+^ exceeded recommended limits at 26.67% of sites, and Mg^2+^ surpassed limits at 16.67% of sites set by (WHO 2022) (Table S2). Calcium naturally occurs in groundwater as CaCO_3_ or CaCl_2_, while magnesium enters from mafic–ultramafic rocks and anthropogenic sources like fertilizers and industries (Razowska-Jaworek 2014). These findings are aligning with previous research (Khanoranga & Khalid 2019; Rapant et al. 2017). Sodium and potassium concentrations ranged from 3 to 190 mgL^–1^ and 0 to 6 mgL^–1^, respectively, with mean values of 61.87 mgL^–1^ and 3.57 mgL^–1^ (Fig. 2a), adhering to permissible limits (WHO 2022). The local lithology impacts Na^+^ and K^+^ concentrations through dissolution, silicate and feldspar weathering, and ion exchange (Khanoranga & Khalid 2019; Ram et al. 2021).

### Spatial variability of physicochemical parameters

**Fig. 3:**
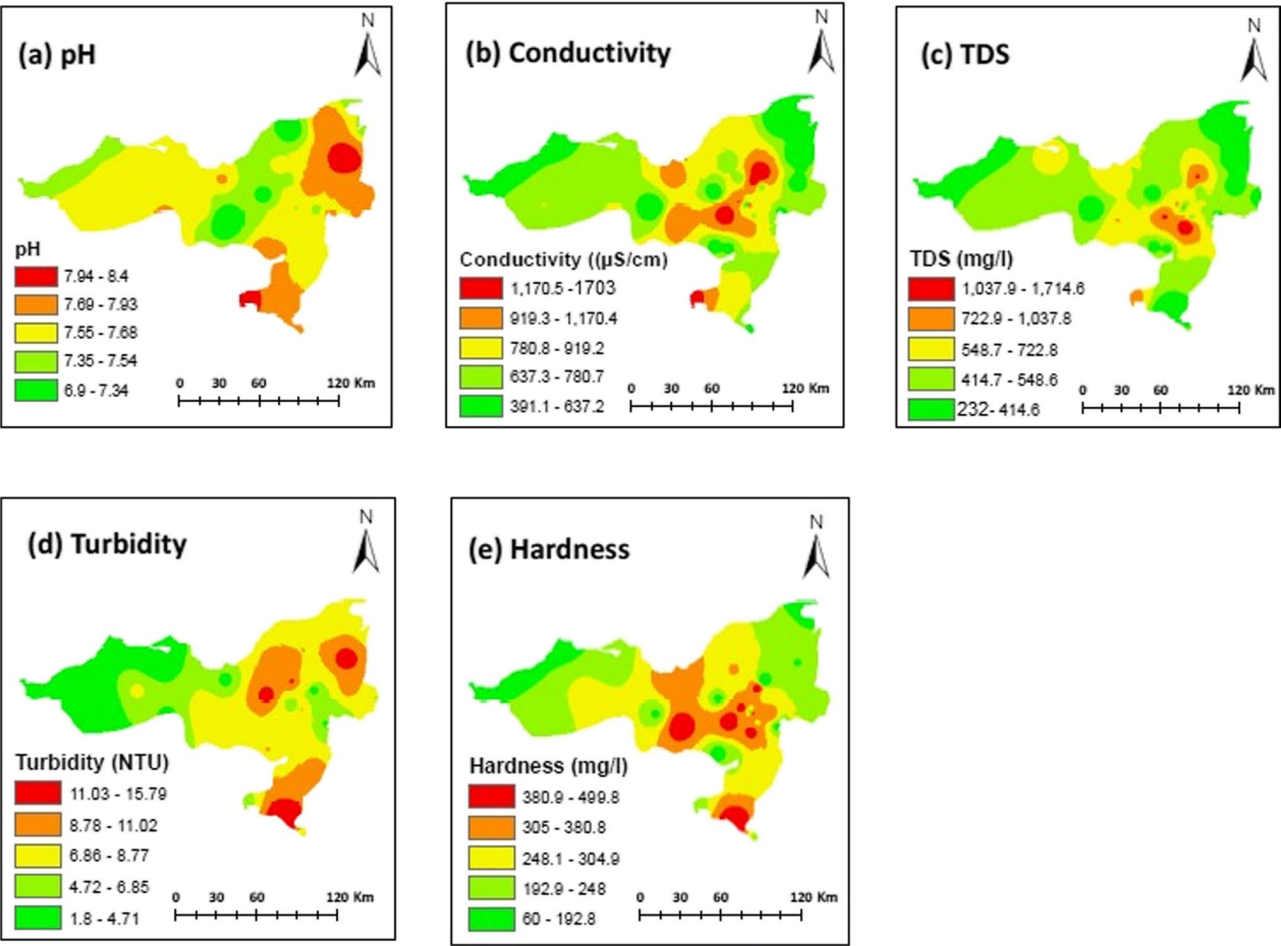
Spatial distribution of physicochemical parameters in the study area

Figure 3a–e shows the spatial distribution of pH, EC, TDS, turbidity, and hardness within the study area. In this study, all physicochemical parameters showed a somewhat consistent distribution pattern, having higher concentrations in eastern regions compared with western area (Fig. 3a–e). Specifically, EC, TDS, and hardness exhibited higher concentrations in the eastern zones, gradually decreasing towards the western areas and northeastern peripheries. Conversely, pH and turbidity displayed elevated levels in the eastern parts, with lower levels observed in the western parts of the study area. Elevated groundwater hardness in the study area was correlated with Na^+^ and SO_4_^2–^, supported by their similar distribution patterns and significant positive correlations (*r*^2^ = 0.89 and 0.92) with hardness. Increased concentrations could be attributed to the presence of limestone, dolomite, calcite, argillite, and gypsum-containing rocks as well as their local mining activities (Jehan et al. 2023; Kumar et al. 2019), which potentially facilitated the release of carbonates and other ions in groundwater. Turbidity, observed at 87% of the sites, surpassed WHO recommended levels. Particularly, higher values were noted in the eastern and city areas of district Zhob, contrasting with lower levels in the western regions (Fig. 3d). Generally, these high concentrations can be attributed to rock weathering and runoff from urban areas.

**Fig. 4:**
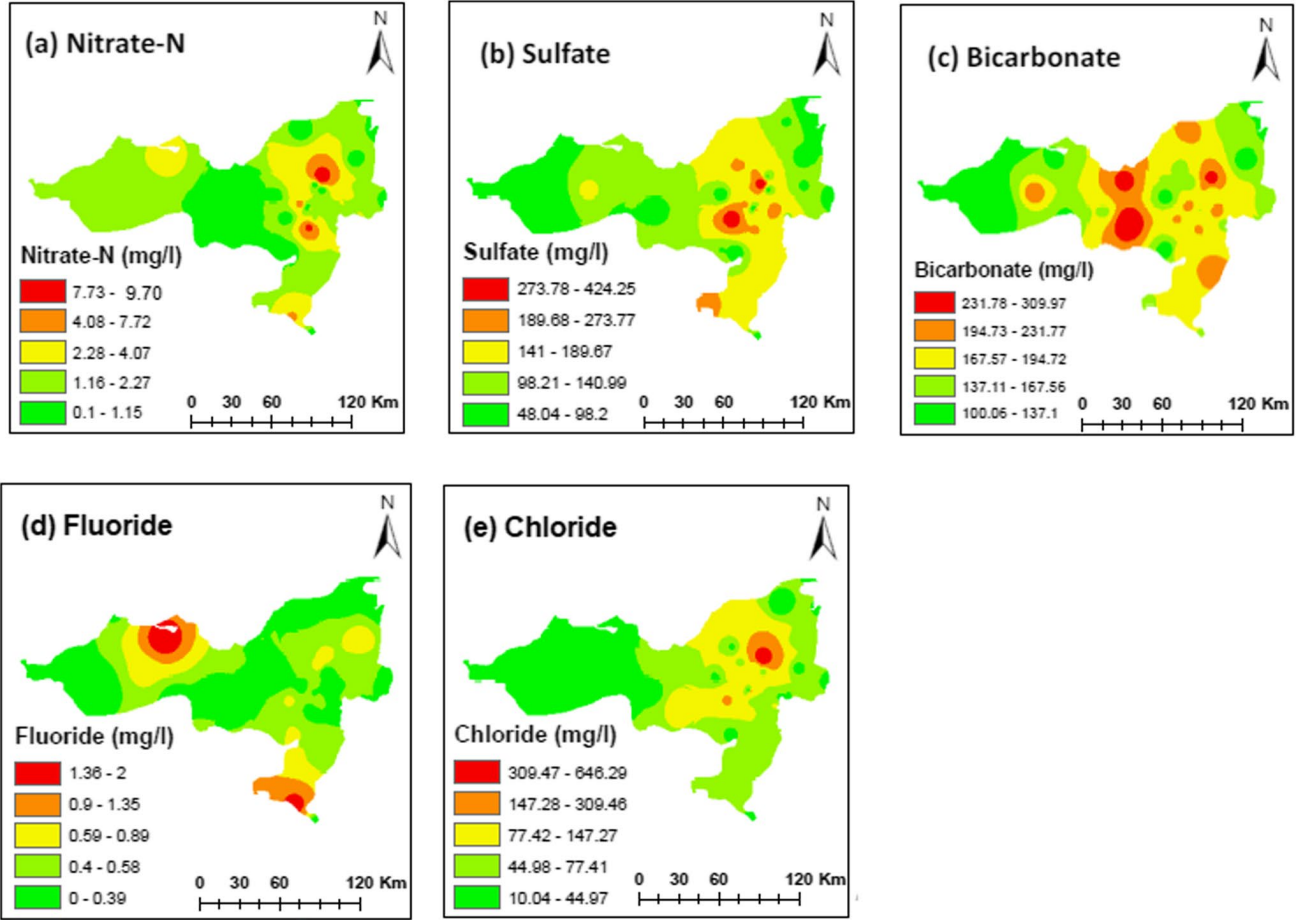
Spatial distribution of studied anions in the study area

Spatially higher concentrations of anions were observed in the central and eastern regions of the study area, with lower values observed towards the western regions (Fig. 4a–e). However, patches of the highest F^–^ concentrations were observed in the northwestern and southeastern parts, along with elevated concentrations around the city (Fig. 4d). The presence of F^–^ primarily arises from geogenic processes involving minerals like fluorite, cryolite, and topaz in rocks formations. NO_3_^–^ and Cl showed a similar distribution pattern, with the highest concentrations near agricultural areas to the east of the city (Fig. 4 a and e). These elevated levels of NO_3_^–^ and Cl^–^ may be attributed to the application of animal manure in agricultural fields and other farming activities. Additionally, Cl^–^ also tends to associate with Ca^2+^, Mg^2+^, and Na^+^, and high Cl^–^ levels can lead to skin issues like dryness and itching. Higher concentrations of bicarbonates were found in areas characterized by high population density and intense human activities, such as urban centers and commercial areas. However, in this study area, HCO_3_^–^ primarily originates from carbon dioxide present in the atmosphere, soil, and carbon-containing rocks, contributing to increased alkalinity (Umar et al. 2013). The spatial distribution of SO_4_^2–^ content extended relatively smoothly across the study area and its presence influences water quality. Notably, higher concentrations of sulfate were observed in and around urban areas, which can be attributed to various human activities such as fossil fuel combustion, municipal waste, and fertilizer use. It is important to note that SO_4_^2–^ concentrations exceeding 1000 mgL^–1^ can be detrimental to plant life.

**Fig. 5:**
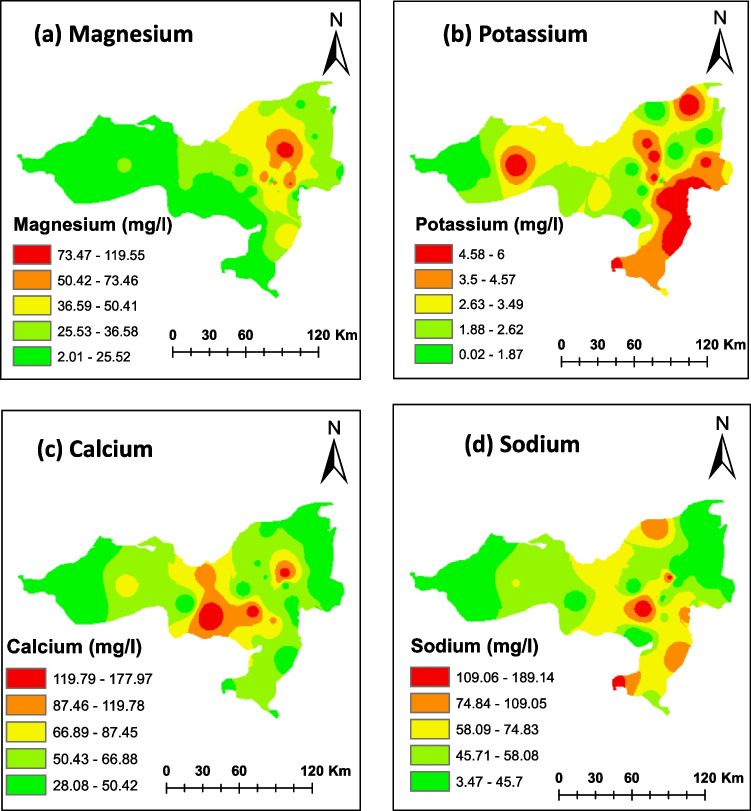
Spatial distributions of studied cations the study area

The enrichment of Mg^2+^ in the northeastern areas may be attributed to fertilizer application within the study area (Fig. 5a). Meanwhile, Ca^2+^ and Na^+^ exhibit concentration in the central and southeastern regions of the study area (Fig. 5 c and d). K^+^ displays a higher presence in the eastern regions, extending towards the western areas through the northern strip, with the lowest concentration observed in the west (Fig. 5b). The distribution of K^+^ is primarily influenced by the presence of K-feldspar and agricultural activities (Umar et al. 2013). These cations’ distribution is significantly influenced by the prevalence of carbonate rocks, limestone, calcite, and dolomite, which have substantial impacts on groundwater quality in the region.

### Correlation matrix

Correlation analysis has been performed on physicochemical parameters of groundwater (Fig. S2). Very high positive correlation (*p* < 0.001) of pH with fluoride indicates that fluoride desorption, mobility, and solubility in drinking water are pH dependent and alkaline water favors fluoride dissolution (Ram et al. 2021). Conversely, pH displayed negative correlations (p < 0.001&0.01) with HCO_3_^–^, Cl^–^, Ca^2+^, and Mg^2+^, indicating their prevalence at lower pH levels. Notably, EC and TDS showed significantly strong positive correlations between themselves as well as with HCO_3_^–^, NO_3_^–^, Cl^–^, Ca^2+^, and Mg^2+^. The correlation between EC and TDS is due to the fact that conductivity depends on TDS, where the primary TDS constituents encompass the aforementioned ionic species (Çankaya et al. 2023; Khanoranga & Khalid 2019). Furthermore, it was evident that HCO_3_^–^dominated alongside contributions from Cl^–^, NO_3_^–^,Ca^2+^, and Mg^2+^ (Rashid et al. 2019; Umar et al. 2013). Significantly high negative correlations of F^–^ with Ca^2+^, Mg^2+^, Cl^–^, NO_3_^–^, and HCO_3_^–^ revealed distinct dissolution mechanisms of these ions compared to F^–^ dissolution (Chandio et al. 2015). Additionally, negative significant correlations with TDS, EC, and hardness revealed that F^–^ does not contribute to TDS, EC, and hardness of groundwater (Chandio et al. 2015). Nitrate has significantly positive correlations with Cl^−^, Mg^2+^, Ca^2+^, HCO_3_^–^,EC, and TDS. Chloride also has significantly positive correlations with Ca^2+^, Mg^2+^, and HCO_3_^–^. As illustrated in the Fig. S2, Ca^2+^, and Mg^2+^ show significantly strong positive correlations with NO_3_^–^, Cl^–^, and HCO_3_^–^, thereby indicating that these ions are weathering products of carbonate rocks and are predominant soluble salts in groundwater (Çankaya et al. 2023). A strong correlation between Ca^2+^ and Mg^2+^ signified the predominance of alkaline earth metal ions over alkali metal ions (Rashid et al. 2019). Hardness exhibited a strong positive correlation with SO_4_^2–^ (0.92), Na^+^ (0.89), and weak with TDS (0.48) and EC (0.48), indicating that hardness in the area is mainly caused by SO_4_^2–^ and Na^+^ (Rashid et al. 2019).

### Drinking water quality

Using WQI model the groundwater quality was evaluated for drinking suitability (Çankaya et al. 2023; Haq & Muhammad 2023; Shanmugasundharam et al. 2023). WQI values ranged from 38.02 to 101.88 with an average of 57.75 in the study area. Assessment of water quality revealed categories including “good,” “poor,” “very poor,” and “undrinkable,” though none were categorized as “excellent.” Specifically, groundwater quality in the study area was classified as “good” at 36.67%, “poor” at 50%, “very poor” at 10%, and “undrinkable” at 3.33% of sites (Table 2 & Fig. S3). Generally, the groundwater quality displayed a subpar status, likely due to elevated levels of TDS, Ca^2+^, Mg^2+^, EC, Cl^−^, SO_4_^2−^, NO_3_^−^, and F^−^, consistent with many previous studies (Çankaya et al. 2023; He et al. 2023; Shanmugasundharam et al. 2023). In this study, only one sample exceeded a WQI value of 100, primarily influenced by NO_3_^−^, Cl^−^, Ca^2+^, and Mg^2+^. Similarly, three sites exhibited very poor water quality, attributed to SO_4_^2−^, TDS, Ca^2+^, and EC. Given that the water from all sampled sites is consumed by Zohb’s residents, it is imperative to take appropriate measures to ensure the provision of safe drinking water to the community.

### Irrigation water quality assessment

Zhob possesses a thriving fruit industry and also embraces diverse crop cultivation, being noteworthy contributors to the region’s economy. The vitality of fruit orchards and agricultural endeavors hinges directly on the availability of suitable irrigation water. Hence, the assessment of irrigation water’s fitness for both crop quality and human health becomes essential. In this pursuit, this study employs a diverse array of indices to thoroughly assess the quality of irrigation water, ensuring it aligns with the criteria essential for sustaining crop quality and safeguarding human health.

#### Sodium percent (Na%)

**Fig. 6:**
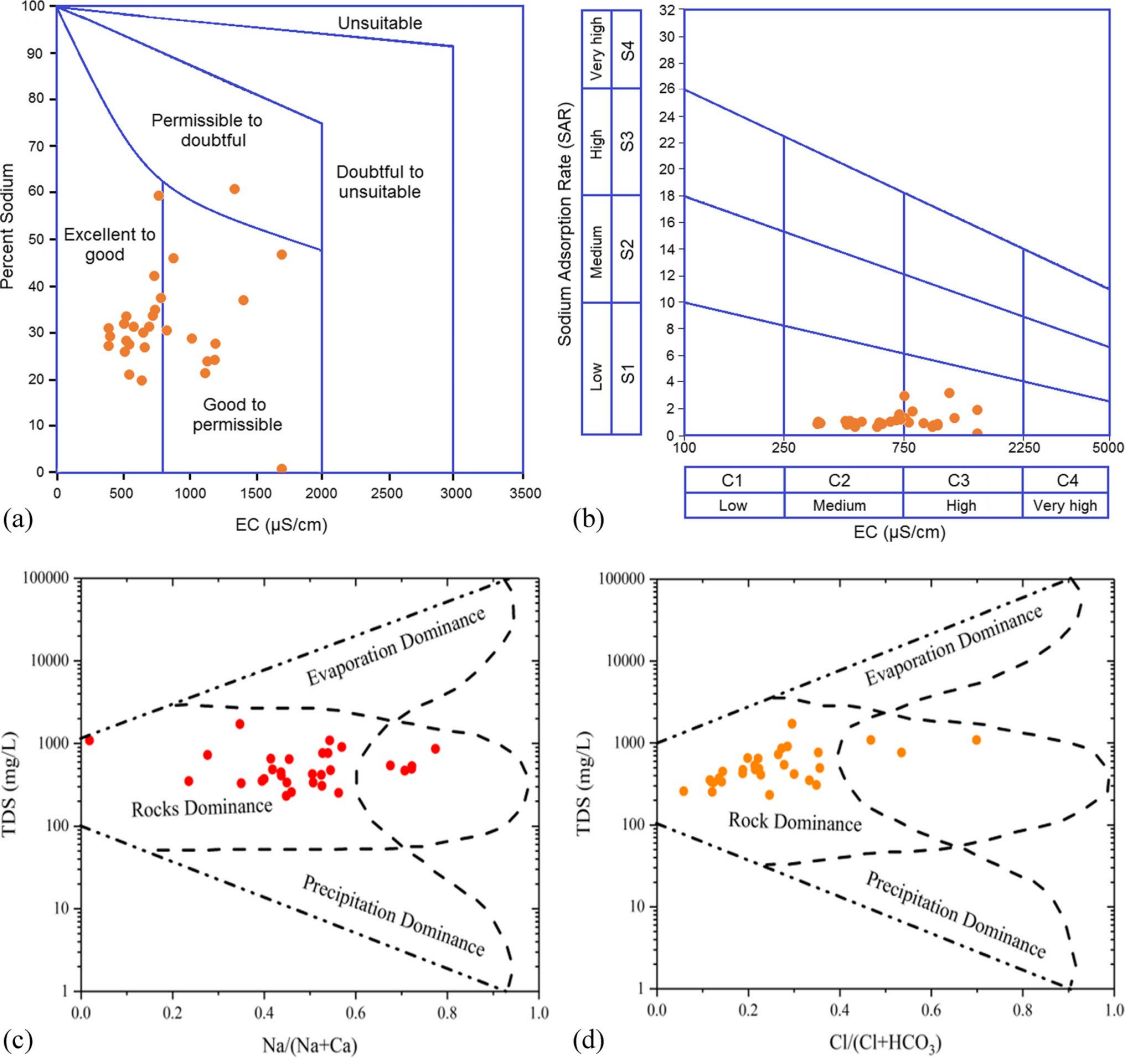
The irrigation water quality demonstrated by **a** Wilcox diagram and **b** USSL diagram. Gibbs diagram representing the potential source of ions in the ground water of study area. TDS versus **c** Na^+^/(Na^+^ + Ca^2+^) and **d** Cl^–^/(Cl^–^ + HCO_3_.^–^)

High sodium levels can harm soil structure, aeration, infiltration, permeability, and thereby plant growth (Panneerselvam et al. 2021). This study found Na% values ranged from 0.72% to 60%, averaging 31.37% (Table 2). Around 6.67% of samples were excellent, 76.67% good, and 16.67% permissible. Slightly high sodium at some points might come from rocks (Khanoranga & Khalid 2019). Overall, the study area’s groundwater is suitable for irrigation, with a lower Na% than in past reports (Khanoranga & Khalid 2019; Umar et al. 2013). The Wilcox diagram plots Na% against EC, resulting in the classification of water samples into five distinct classes, ranging from excellent to unsuitable (Yang, et al. 2023). Wilcox diagram (Fig. 6a) shows groundwater mainly in excellent to good (63.33%) and good to permissible (33.33%) classes. Thus, the study area’s groundwater is safe for irrigation (Çankaya et al. 2023).

#### Sodium adsorption ratio (SAR)

SAR is crucial for assessing irrigation water’s suitability. High SAR can impact soil structure, compaction, infiltration, and crop growth due to increased Na^+^ content (Chakraborty et al. 2022; Samtio et al. 2022). In this study SAR values ranged from 0.01 to 2.98 meqL^–1^, averaging 0.98 meqL^–1^, with only 26.67% exceeding 1.0 meqL^–1^ (Table 2). The groundwater SAR values were safe (SAR < 10), classifying all samples as excellent for irrigation (Çankaya et al. 2023; Lanjwani et al. 2022). Considering SAR, our study’s water quality aligns with Ghizer’s water quality (Haq & Muhammad 2023) and is better than Rawalpindi’s water quality (Khan et al. 2019). The USSL diagram corroborated findings, plotting EC (salinity) against SAR (alkalinity) (Fig. 6b). Samples mainly fell in C2 and C3 dominance, with 70% in C2 and 30% in C3, indicating that moderate salinity and few samples have high salinity. Furthermore, C2S1 class (medium salinity, low alkalinity) had the most samples, followed by C3S1 class (high salinity, low alkalinity). Therefore, groundwater can be used for irrigation with moderate leaching (Yang et al. 2023). Long-term use without proper leaching can accumulate harmful Na^+^ levels, harming soil and crop yield. Planting halophytes with soil management is recommended to avoid salinity issues (Çankaya et al. 2023).

#### Residual sodium carbonate (RSC)

RSC determines irrigation water suitability via carbonate and bicarbonate ratio. Negative RSC indicates excess sodium, displacing calcium and magnesium, with calcium precipitating as CO_2_. Positive RSC implies elevated calcium and magnesium from HCO_3_^–^ reaction to form bicarbonate of calcium/magnesium (Khanoranga & Khalid 2019). This study showed RSC ranging from –13.12 to –0.79 meqL^–1^ (average –3.15 meqL^–1^) (Table 2). Negative RCS suggests fewer anions (CO_3_^2–^ + HCO_3_^–^) than cations (Ca^2+^ + Mg^2+^) in groundwater, classifying it as good for irrigation (Çankaya et al. 2023).

#### Kelley index (KI)

Kelley index assesses Na^+^ toxicity of groundwater and its suitability for irrigation. Our study revealed KI values ranging from 0.01 to 1.49 with an average of 0.49. Around 93% of groundwater samples had KI < 1.0, signifying low Na^+^ levels and suitability for irrigation (Table 2). However, 7% had KI > 1.0, indicating high Na^+^ content and unsuitability for irrigation (Chakraborty et al. 2022). Our KI results showcased improved groundwater quality compared to prior data from the Zhob River Basin (Umar et al. 2013). Overall, the groundwater is classified as suitable for irrigation (Table 2).

#### Permeability index (PI)

Extended use of Na^+^, Ca^2+^, Mg^2+^ and HCO_3_^−^ rich water can detrimentally impact soil permeability, aeration, and compaction, subsequently reducing crop yield (Rufino et al. 2019). Therefore, the PI appeared to be a robust tool to evaluate water suitability for irrigation (Yang et al. 2023). This study found PI values ranging from 13.45 to 74.00%, with an average of 49.11% (Table 2). PI results categorized 3% of samples as excellent (Class I) and 97% as suitable (Class II) for irrigation. This suggests that groundwater from all sampling sites is appropriate for irrigation purposes (Khan et al. 2023).

#### Magnesium hazard (MH)

MH in groundwater exhibited a range of 4.10 to 81.70, with a mean of 46.11% (Table 2). Among the samples, 46.67% were deemed suitable (MH < 50), while 53.33% were considered unsuitable (MH > 50) for irrigation in line with previous studies (Khanoranga & Khalid 2019; Panneerselvam et al. 2021). Elevated magnesium concentration often results from sodium ion exchange in groundwater (Khanoranga & Khalid 2019). Parent rock weathering and the dissolution of calcite and dolomite significantly contribute to the abundance of magnesium ions (Panneerselvam et al. 2021). Unlike other irrigation water indices, MH highlighted compromised groundwater quality that might impair crop yield. Although Mg^2+^ is a vital plant nutrient, its high concentration in water can elevate soil alkalinity, potentially reducing crop productivity (Khan et al. 2023). Therefore, addressing MH is crucial to safeguard crop health in the study area.

#### Potential salinity (PS)

Potential salinity (PS) is the sum of Cl– and ½ SO_4_^2–^ and is a widely applied parameter to assess groundwater suitability for irrigation. Water having PS < 3 is considered suitable for agriculture; if PS > 3, it is deemed unsuitable (Çankaya et al. 2023). Within the study area, PS ranged from 1.18 to 19.34 meqL^–1^, with a mean of 3.93 meqL^–1^ (Table 2). It was observed that 43.33% of samples were deemed suitable, while 56.67% were considered unsuitable for agricultural use.

### Gibbs diagram

Hydrochemical data were plotted on a Gibbs diagram (Fig. 6 c and d) to assess the influence of atmospheric precipitation, rock weathering, and sea evaporation on the groundwater chemistry of the study area (Çankaya et al. 2023; Jehan et al. 2023; Khan et al. 2023; Muhammad & Ullah 2022). The collected water samples exhibited moderate TDS levels (between 100 and 1000 mgL^–1^), along with low Na^+^/(Na^+^  + Ca^2+^) and Cl^−^ /(Cl^−^  + HCO_3_^−^) ratios (< 0.5), indicating a prevailing influence of rock characteristics. Consequently, the hydrochemistry is primarily governed by rock weathering, contributing to the suboptimal groundwater quality in the study area. The dominance of silicate weathering and soft evaporite dissolution was further confirmed by bivariate plots of Ca^2+^/Na^+^ vs HCO_3_^–^/Na^+^ and Ca^2+^/Na^+^ vs Mg^2+^/Na^+^ (Fig. S4). Numerous previous studies have reported analogous results (Çankaya et al. 2023; Muhammad & Ullah 2022; Vaiphei & Kurakalva 2021). The average Ca^2+^ + Mg^2+^/Na^+^ + K^+^ molar ratio (2.32) surpassing typical crustal silicates ratio (1.0) signifies a higher contribution of cations from alumino-sillicate weathering compared to carbonates (Fakharian & Narany 2016). Moreover, the lower Na^+^/(Na^+^ + Ca^2+^) and Cl^−^/(Cl^−^ + HCO_3_^–^) ratios suggest the presence of mafic–ultramafic rocks, which typically exhibit elevated levels of sodium and chloride.

### Hydrogeochemical facies

**Fig. 7:**
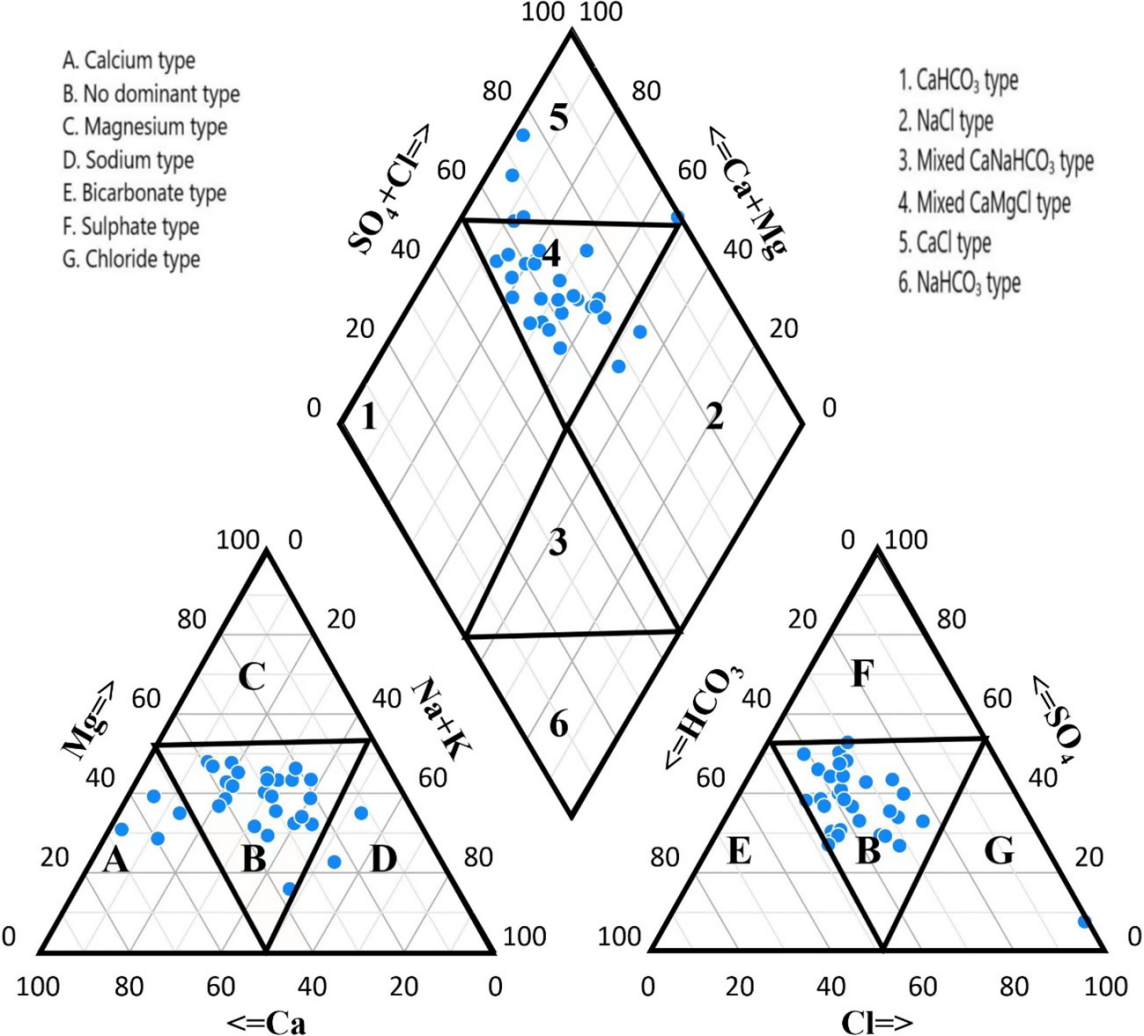
Hydrochemical facies of groundwater in the study area

Based on the observed mean concentrations, the major cations at all sampling sites were in the order of Ca^2+^  > Na^+^  > Mg^2+^  > K^+^. Similarly, the mean concentrations of major anions were in the order of HCO_3_^−^  > SO_4_^2−^  > Cl^−^  > NO_3_^−^  > F^–^ > CO_3_^2–^. These findings align with previous research (Çankaya et al. 2023; Umar et al. 2013) which demonstrated the predominance of Ca^2+^ as the most abundant cation and HCO_3_^−^ as the most prevalent anion. The classification of water types was graphically represented using a Piper diagram (Fig. 7), which enabled the assessment of hydrogeochemical facies and diverse water categories of the groundwater (Salem et al. 2023; Sinha et al. 2023; Yang et al. 2023). In the cationic plot, the majority (80%) of groundwater samples were situated within zone B, signifying the absence of a dominant water type. Calcium and sodium accounted for only 13.33% and 6.667% of the total samples, respectively. In contrast, the anionic plot displayed 96.67% of samples within zone B, signifying a lack of dominance, with chloride-type water representing a mere 3.3% of samples. Analysis of the diamond-shaped chart revealed that 76.67% of the samples were categorized in zone 4, indicating that a mixed Ca–Mg–Cl water type prevails in the study area (Ram et al. 2021). The occurrence of Ca–Cl and Na–Cl water types was noted in 13.33% and 6.67% of samples, respectively. The hydrofacies, such as Na–Cl and Ca–Cl, could arise due to cation exchange processes and water–rock interactions, encompassing the dissolution of halite and calcium carbonate rocks (Khanoranga & Khalid 2019). In conclusion, the dominant water type in the study area is determined to be mixed Ca–Mg–Cl, a finding corroborated by (Ghoraba & Khan 2013) in previous research. The prevalence of Ca^2+^ and Mg^2+^ suggests the predominance of sedimentary rocks and limestone, as affirmed by (Umar et al. 2013). The local geological composition such as sedimentary rocks including limestone, dolomite, argillites, and sandstone are deciding the hydrological facies.

### Human health risk assessment (HHRA)

Assessment of drinking water quality, especially regarding NO_3_^–^ and F^–^ exposure through ingestion and dermal contact, was conducted to evaluate non-cancer health risks among children, adult females, and adult males (Fig. 2b & Table S3) (Selvam et al. 2023; Yang et al. 2022). Consistent with previous studies, ingestion-based Hazard Quotient (HQ) values exceeded dermal HQ values across all subpopulations (Jadoon et al. 2021; Varol & Tokatlı, 2023). Approximately 56.67%, 10%, and 6.67% of groundwater samples exhibited ingestion HQ values exceeding the threshold value (1.0) for children, adult females, and adult males, respectively. Conversely, dermal HQ remained below 1 for all subpopulations at all sites (Fig. 2b). The total hazard quotient (THQ) calculated for NO_3_^–^ and F^–^ indicated that THQ for NO_3_^–^ did not surpass 1 at any site for all subpopulations. For F^–^, THQ > 1 was observed at 50%, 10%, and 6.67% sites for children, adult females, and adult males (Fig. 2b). These findings emphasize the significance of the ingestion pathway and F^–^ exposure as key contributors to potential non-cancer risk (Table S3 & Fig. S5) (Çankaya et al. 2023). Moreover, children faced the highest risk, followed by adult females and adult males (Varol & Tokatlı, 2023). Average hazard index (HI) values were 1.24.E + 00, 5.94.E-01, and 4.36.E-01 for children, adult females, and adult males, respectively (Fig. 2b). Given that average HI values for children exceeded risk thresholds, this subpopulation is susceptible to characteristic non-cancer risks. This study underscores that children face higher non-cancer risks compared to both adult groups, consistent with previous reports (Jadoon et al. 2021; Selvam et al. 2023; Varol & Tokatlı, 2023; Yang et al. 2022). Children are exposed to potential non-cancer risks at 56.67% of sites, while adult females and males face such risks at 10% and 6.67% of sites, respectively.

The HI values across all populations exhibited a significantly strong positive correlation with F^–^ concentrations and a non-significant correlation with NO_3_^–^ concentrations, indicating that F^–^ is the primary risk factor for residents (Fig. S5) (Qu et al. 2022). Children’s increased vulnerability to groundwater NO_3_^–^ and F^–^ pollutants could be attributed to their underdeveloped metabolism and lower body weight (Qu et al. 2022).

Exceeding permissible groundwater concentrations of NO_3_^–^ and F^–^ can lead to health complications like blue baby syndrome, esophageal and gastric cancers, and dental and skeletal fluorosis (Vaiphei & Kurakalva 2021). This study’s outcomes suggest that along with other parameters, NO_3_^–^ and F^–^ pollution in groundwater could arise from water–rock interactions. The establishment of robust groundwater quality monitoring networks is crucial for effective groundwater management in the study area.

## Conclusions

The study highlights the vital role of groundwater for domestic and agricultural needs in the semi-arid study area. The evaluation of water quality against established standards presents a varied outlook. While certain parameters meet WHO guidelines, the presence of contaminants is concerning. pH, EC, and TDS generally stay within limits, though exceptions exist. Elevated turbidity levels raise aesthetic and health worries. Dominant ions include bicarbonates, sulfates, chlorides, calcium, sodium, and magnesium. Although ion compositions usually align with norms, occasional excesses occur. Many physicochemical parameters surpass WHO limits, emphasizing the need for water quality enhancements. Drinking water quality varies from good to undrinkable, with no samples in the excellent range, and a notable portion falls into the poor category. TDS, calcium, magnesium, EC, and chloride contributed to drinking water quality deterioration. Irrigation indices suggest groundwater use for irrigation without initial treatment, but prolonged use without soil management may lead to sodium and magnesium hazards. Hydrochemical analysis attributes groundwater composition to rock-water interaction, primarily from silicate weathering and cation exchange. The hydrochemical facies lack a dominant water type, with mixed Ca–Mg–Cl being common, along with Na–Cl and Ca–Cl types. Fluoride poses non-cancer risks, particularly for children and adult females, underscoring the need for caution in using groundwater for drinking. In conclusion, the study emphasizes groundwater’s significance in the study area and its intricate water quality. Addressing highlighted issues is pivotal for safe and sustainable domestic and agricultural use of groundwater in the region.

## Recommendation and limitations

Based on this study’s findings, it was observed that TDS, Ca^2+^, Mg^2+^, EC, Cl^−^, SO_4_^2−^, NO_3_^−^, and F^−^ have degraded drinking water quality, while magnesium hazard and potential salinity have affected irrigation water. Prolonged use of such compromised water poses risks to human health and soil quality, including compaction and salinity. Therefore, special attention to children is recommended to prevent future health issues and soil degradation associated with this contaminated water. This can be achieved through water treatment, regular monitoring, and construction of barriers around wells. Additional strategies include reducing soil salinity, community engagement, pre-treatment of industrial effluents, and constructing sewage treatment plants to ensure public health and improve soil quality and crop yield.

While this study offers a comprehensive assessment of drinking and irrigation water quality and associated human and environmental health risks, it has limitations. Funding constraints restricted the sample size, potentially compromising its representativeness. Additionally, the study’s reliance on a single sampling event suggests the need for seasonal evaluations to understand water quality parameters and their responses to seasonal changes more comprehensively. Future research should consider incorporating machine learning algorithms to enhance findings and predictions. Furthermore, the study may have provided only a snapshot of groundwater quality at a specific point in time, highlighting the importance of considering temporal variations for a comprehensive understanding of water quality dynamics and long-term trends.

### Supplementary Information

Below is the link to the electronic supplementary material.

Supplementary file1 (DOCX 4373 KB)

## Data Availability

All data analyzed during this study are included in this published article and are not publicly available but may be obtained from the corresponding author on reasonable request.
