# Supplementary material for: Assessment of hydrochemical characteristics, health risks and quality of groundwater for drinking and irrigation purposes in a mountainous region of Pakistan
Source: Environ Sci Pollut Res Int. 2024 Jun 25;31(31):43967–86. doi: 10.1007/s11356-024-34046-7 (PMC11252193; doi:10.1007/s11356-024-34046-7)
Supplement: Supplementary file 1 — Supplementary file1 (DOCX 4373 KB) [file 11356_2024_34046_MOESM1_ESM.docx]

**Supplementary Material**

**Supplementary Text S1**

**Sodium percentage (Na%) or sodium hazard**

The Na% is used in classifying water for irrigation purpose and is calculated using the formula (eq. 2) given below, where all the concentrations are in meqL^–1^.

$Na\%=\frac{Na^{+}+ K^{+}}{Ca^{2+} + Mg^{2+} + Na^{+}+ K^{+}}x 100$ (1)

Na^+^ is an important parameter that plays a crucial role in classifying water resources for irrigation purposes. The classification of water is based on Na% and is defined as as excellent (< 20%), good (20–40%), permissible (40–60%), doubtful (60–80%) and unsuitable (> 80%)(Rawat et al., 2018).

**Sodium adsorption ratio (SAR)**

The SAR is a proportional measure of the concentration of Na^+^ ion compared to Ca^2+^ and Mg^2+^ ions within a water sample. SAR is employed to assess the likelihood of Na^+^ accumulation in the soil, predominantly through water movement, often at the expense of Ca^2+^, Mg^2+^ and K^+^ ions due to the consistent utilization of sodic water. It is formulated as Eq. (1):

$SAR=\frac{Na^{+}}{\sqrt{\frac{Ca^{2+}+Mg^{2+}}{2}}}$ (2)

where, cationic concentrations are in milliequivalents per litre (meqL^–1^ ).

Based on SAR values, irrigation water is categorized into four classes: SAR < 10 (ideal or excellent), 10–18 (good), 18–26 (doubtful) and > 26 (unsuitable). The SAR also influences water percolation rate within the soil, making a low SAR value highly desirable. Elevated sodium levels in water can potentially yield detrimental effects in most soils, necessitating special water and soil management practices, often involving gypsum application.

**Residual sodium carbonate (RSC)**

The RSC represents as the amount of sodium carbonate (NaCO_3_) and sodium bicarbonate (NaHCO_3_) present in the irrigation water. When the concentration of CO_3_^2−^ and HCO_3_^−^ ions exceeds the concentrations of Ca^2+^ and Mg^2+^ ions, it leads to the precipitation of Ca^2+^ and Mg^2+^ (Raghunath 1987). If the carbonates are less than alkaline earths (Ca^2+^ + Mg^2+^), it indicates the presence of residual NaCO_3_. Generally, RSC is expressed as meqL^–1^ of NaCO_3_. An excessive presence of CO_3_^2−^and HCO_3_^−^ triggers the precipitation of soil Ca^2+^ and Mg^2+^, impairing soil structure and potentially activating soil sodium. RSC is expressed as Eq. ([3](https://link.springer.com/article/10.1007/s13201-018-0866-8#Equ2)):

$RSC=\left( HCO_{3}^{-} +CO_{3}^{2-} \right) -\left( Ca^{2+}+Mg^{2+} \right)$ (3)

Based on RSC values, sodium hazard is classified into three classes: RSC < 1.25 (low), 1.25–2.5 (medium), and > 2.5 (high). A higher RSC value in water leads to an increased adsorption of sodium in soil (Eaton, 1950). Water with RSC < 1.25 meqL^–1^ is considered suitable for irrigation, while RSC values exceeding 2.5 meqL^–1^ are deemed unsuitable. RSC values greater than 5 meqL^–1^ are harmful to plant growth. A negative RSC reveals an excess of Ca^2+^ and Mg^2+^ concentrations. A positive RSC denotes the potential presence of Na^+^ in soil.

**Kelly’s index (KI)**

Kelly’s index is used to valuate the quality and categorization of water for irrigation purpose based on the Na^+^ concentration in relation to Ca^2+^ and Mg^2+^. KI is calculated using Eq. (4), where all the concentrations were in meqL^–1^.

$KI=\frac{\left( Na^{+} \right)}{\left( Ca^{2+} + Mg^{2+} \right)}$ (4)

A KI > 1 indicates an excess level of Na^+^ in waters. Therefore, water with a KI ≤ 1 is advisable for irrigation, while water with a KI ≥ 1 is not recommended due to the potential alkali hazards (Rawat et al., 2018).

**Permeability index (PI)**

The PI serves as an indicator for assessing the appropriateness of water for irrigation purposes. The soil’s permeability is influenced by the prolonged use of irrigation water with elevated salt concentration, leading to alterations caused by Na^+^, Ca^2+^, Mg^2+^ and HCO_3_^−^ ions within the soil (Ishaku, et al. 2011). Developed by Doneen (1964), the PI evaluates water’s capacity to move within the soil, thereby determining the suitability of various water sources for irrigation. The formula is expressed through Equation (5):

$PI=\frac{Na^{+}+\sqrt{\mathrm{HC}O_{3}^{-}}}{Ca^{2+}+ Mg^{2+}+Na^{+}} x 100$ (5)

The PI can be categories into three classes: class I (> 75%, suitable), class II (25–75%, good) and class III (< 25%, unsuitable). Water falling within class I and class II is recommended for irrigation.

**Magnesium hazard (MH)**

The presence of magnesium in groundwater has a significant impact on soil quality, leading to an increase in soil alkalinity and a subsequent reduction in crop yield(Khanoranga & Khalid, 2019). Szabolcs and Darab ([1964](https://link.springer.com/article/10.1007/s13201-018-0866-8#ref-CR58)) formulated MH values for assessing irrigation water, which can be calculated (using ion concentrations in meqL^–1^) using Eq. (6):

$MH=\frac{\mathrm{Mg}^{2+}}{(Ca^{2+}+ Mg^{2+})} x 100$ (6)

If MH < 50 then it is considered safe for irrigation use; however, if exceeds 50, it is considered unsuitable (Chidambaram et al. 2022).

**Potential salinity (PS)**

The PS index, developed by Doneen (1964), is another water quality parameter-based metric used for classifying water for irrigation purposes. A PS < 3 meqL^–1^ indicates the water’s suitability for irrigation. The distribution of the PS in the study area is generated using the following Equation (7):

$PS=Cl^{-}+0.5 x SO_{4}^{2-}$ (7)

**Table S1.** Summary of variable used to calculate Human Health Risk Assessment (HHRA;(Qu et al., 2022)).

| **Variable for risk analysis** | **Unit** | **Values** | | |
| --- | --- | --- | --- | --- |
|  |  | **Children** | **Female** | **Male** |
| Pollutant concentration in water (C_w_) | mg L^–1^ | - | - | - |
| Ingestion rate (IR_ing_)* | L day^–1^ | 1.25 | 2.2 | 2.2 |
| Exposure frequency (EF) | Day Year^–1^ | 365 | 365 | 365 |
| Exposure duration (ED) | Year | 6 | 30 | 30 |
| Body weight (BW) | kg | 15 | 55 | 75 |
| Average time (AT) | Day | 2190 | 10950 | 10950 |
| Skin surface area (S_a_) | - | 6597.01 | 15475.85 | 18742.36 |
| Skin permeability coefficient (K_p_) | cm h^–1^ | 0.001 | 0.001 | 0.001 |
| Contact duration (T) | h day^–1^ | 0.4 | 0.4 | 0.4 |
| Exposure frequency of daily dermal contact (EV) | - | 1 | 1 | 1 |
| Unit conversion factor (CF) | L cm^–3^ | 0.001 | 0.001 | 0.001 |
| Oral reference dose for F^–^ (RfD oral (F^–^)) | mg kg^–1^ day^–1^ | 0.04 | 0.04 | 0.04 |
| Oral reference dose for NO_3_^–^ (RfD oral (NO_3_^–^)) | mg kg^–1^ day^–1^ | 1.6 | 1.6 | 1.6 |
| Gastrointestinal absorption factor ABS_gi_ | - | 1 | 1 | 1 |

*Islam et al., (2020)

**Table S2.** Physicochemical parameters assessment in the groundwater of the District Zhob and their comparison with the World Health Organization (WHO). The values of EC are in μScm^–1^, Turbidity in NTU, and the remaining parameters are in mgL^–1^.

| Parameters | Maximum permissible limit (WHO 2011) | Mean values in Zhob | Number of samples exceeding WHO standard | Percentage of samples exceeding WHO standard |
| --- | --- | --- | --- | --- |
| pH | 6.5-8.5 | 7.64 | 0 | 0.00% |
| EC (μScm^–1^) | 1000 | 830.13 | 9 | 30.00% |
| TDS (mgL^–1^) | 1000 | 562.83 | 3 | 10.00% |
| Na^+^ (mgL^–1^) | 200 | 61.87 | 0 | 0.00% |
| Mg^2+^ (mgL^–1^) | 50 | 34.89 | 5 | 16.67% |
| NO_3_^-^ (mgL^–1^) | 45 | 2.61 | 0 | 0.00% |
| Ca^2+^ (mgL^–1^) | 75 | 65.00 | 8 | 26.67% |
| F^–^ (mgL^–1^) | 1.5 | 0.55 | 2 | 6.67% |
| Cl^-^ (mgL^–1^) | 250 | 81.43 | 1 | 3.33% |
| Hardness (mgL^–1^) | 500 | 285.80 | 0 | 0.00% |
| K^+^ (mgL^–1^) | 12 | 3.57 | 0 | 0.00% |
| HCO_3_^-^ (mgL^–1^) | 500 | 183.33 | 0 | 0.00% |
| Turbidity (NTU) | 5 | 7.99 | 25 | 83.33% |
| SO_4_^2-^ (mgL^–1^) | 250 | 156.67 | 4 | 13.33% |

**Table S3.** Total hazard quotient and non-cancerous health risk to the residents at all sampling sites from district Zhob, Pakistan

| **Exposure pathway wise total hazard quotient (THQ)** | | | | | | **Chemical species wise total hazard quotient (THQ)** | | | | | | **Non-cancerous risk** | | |
| --- | --- | --- | --- | --- | --- | --- | --- | --- | --- | --- | --- | --- | --- | --- |
| **THQ children** | | **THQ adult female** | | **THQ adult male** | | **THQ children** | | **THQ adult female** | | **THQ adult male** | | **Hazard Index (HI)** | | |
| **Ingestion** | **Dermal** | **Ingestion** | **Dermal** | **Ingestion** | **Dermal** | **NO_3_^–^** | **F^–^** | **NO_3_^–^** | **F^–^** | **NO_3_^–^** | **F^–^** | **Children** | **Female** | **Male** |
| 1.79.E+01 | 3.78.E-03 | 8.60.E-01 | 2.42.E-03 | 6.31.E-01 | 2.15.E-03 | 3.13.E-01 | 1.48.E+00 | 1.50.E-01 | 7.12.E-01 | 1.10.E-01 | 5.22.E-01 | **1.80.E+00** | **8.62.E-01** | **6.33.E-01** |
| 1.40.E+00 | 2.95.E-03 | 6.70.E-01 | 1.89.E-03 | 4.91.E-01 | 1.67.E-03 | 0.00.E+00 | 1.40.E+00 | 0.00.E+00 | 6.72.E-01 | 0.00.E+00 | 4.93.E-01 | **1.40.E+00** | **6.72.E-01** | **4.93.E-01** |
| 8.75.E-01 | 1.85.E-03 | 4.20.E-01 | 1.18.E-03 | 3.08.E-01 | 1.05.E-03 | 0.00.E+00 | 8.77.E-01 | 0.00.E+00 | 4.21.E-01 | 0.00.E+00 | 3.09.E-01 | **8.77.E-01** | **4.21.E-01** | **3.09.E-01** |
| 1.67.E+00 | 3.52.E-03 | 8.00.E-01 | 2.25.E-03 | 5.87.E-01 | 2.00.E-03 | 0.00.E+00 | 1.67.E+00 | 0.00.E+00 | 8.02.E-01 | 0.00.E+00 | 5.89.E-01 | **1.67.E+00** | **8.02.E-01** | **5.89.E-01** |
| 9.17.E-01 | 1.94.E-03 | 4.40.E-01 | 1.24.E-03 | 3.23.E-01 | 1.10.E-03 | 0.00.E+00 | 9.19.E-01 | 0.00.E+00 | 4.41.E-01 | 0.00.E+00 | 3.24.E-01 | **9.19.E-01** | **4.41.E-01** | **3.24.E-01** |
| 3.13.E-01 | 6.60.E-04 | 1.50.E-01 | 4.22.E-04 | 1.10.E-01 | 3.75.E-04 | 0.00.E+00 | 3.13.E-01 | 0.00.E+00 | 1.50.E-01 | 0.00.E+00 | 1.10.E-01 | **3.13.E-01** | **1.50.E-01** | **1.10.E-01** |
| 1.10.E+00 | 2.33.E-03 | 5.30.E-01 | 1.49.E-03 | 3.89.E-01 | 1.32.E-03 | 0.00.E+00 | 1.11.E+00 | 0.00.E+00 | 5.31.E-01 | 0.00.E+00 | 3.90.E-01 | **1.11.E+00** | **5.31.E-01** | **3.90.E-01** |
| 5.63.E-01 | 1.19.E-03 | 2.70.E-01 | 7.60.E-04 | 1.98.E-01 | 6.75.E-04 | 0.00.E+00 | 5.64.E-01 | 0.00.E+00 | 2.71.E-01 | 0.00.E+00 | 1.99.E-01 | **5.64.E-01** | **2.71.E-01** | **1.99.E-01** |
| 1.40.E+00 | 2.95.E-03 | 6.70.E-01 | 1.89.E-03 | 4.91.E-01 | 1.67.E-03 | 0.00.E+00 | 1.40.E+00 | 0.00.E+00 | 6.72.E-01 | 0.00.E+00 | 4.93.E-01 | **1.40.E+00** | **6.72.E-01** | **4.93.E-01** |
| 1.21.E+00 | 2.55.E-03 | 5.80.E-01 | 1.63.E-03 | 4.25.E-01 | 1.45.E-03 | 0.00.E+00 | 1.21.E+00 | 0.00.E+00 | 5.82.E-01 | 0.00.E+00 | 4.27.E-01 | **1.21.E+00** | **5.82.E-01** | **4.27.E-01** |
| 4.17.E-01 | 8.80.E-04 | 2.00.E-01 | 5.63.E-04 | 1.47.E-01 | 5.00.E-04 | 0.00.E+00 | 4.18.E-01 | 0.00.E+00 | 2.01.E-01 | 0.00.E+00 | 1.47.E-01 | **4.18.E-01** | **2.01.E-01** | **1.47.E-01** |
| 2.08.E-02 | 4.40.E-05 | 1.00.E-02 | 2.81.E-05 | 7.33.E-03 | 2.50.E-05 | 2.09.E-02 | 0.00.E+00 | 1.00.E-02 | 0.00.E+00 | 7.36.E-03 | 0.00.E+00 | **2.09.E-02** | **1.00.E-02** | **7.36.E-03** |
| 1.75.E+00 | 3.69.E-03 | 8.40.E-01 | 2.36.E-03 | 6.16.E-01 | 2.10.E-03 | 2.09.E-02 | 1.73.E+00 | 1.00.E-02 | 8.32.E-01 | 7.36.E-03 | 6.11.E-01 | **1.75.E+00** | **8.42.E-01** | **6.18.E-01** |
| 6.93.E-01 | 1.46.E-03 | 3.33.E-01 | 9.36.E-04 | 2.44.E-01 | 8.31.E-04 | 1.51.E-01 | 5.43.E-01 | 7.27.E-02 | 2.61.E-01 | 5.33.E-02 | 1.91.E-01 | **6.94.E-01** | **3.33.E-01** | **2.45.E-01** |
| 6.93.E-01 | 1.46.E-03 | 3.33.E-01 | 9.36.E-04 | 2.44.E-01 | 8.31.E-04 | 1.10.E-01 | 5.85.E-01 | 5.26.E-02 | 2.81.E-01 | 3.86.E-02 | 2.06.E-01 | **6.94.E-01** | **3.33.E-01** | **2.45.E-01** |
| 1.18.E+00 | 2.48.E-03 | 5.65.E-01 | 1.59.E-03 | 4.14.E-01 | 1.41.E-03 | 1.15.E-01 | 1.06.E+00 | 5.52.E-02 | 5.11.E-01 | 4.05.E-02 | 3.75.E-01 | **1.18.E+00** | **5.67.E-01** | **4.16.E-01** |
| 4.22.E-01 | 8.91.E-04 | 2.03.E-01 | 5.70.E-04 | 1.49.E-01 | 5.06.E-04 | 8.87.E-02 | 3.34.E-01 | 4.26.E-02 | 1.60.E-01 | 3.13.E-02 | 1.18.E-01 | **4.23.E-01** | **2.03.E-01** | **1.49.E-01** |
| 9.90.E-01 | 2.09.E-03 | 4.75.E-01 | 1.34.E-03 | 3.48.E-01 | 1.19.E-03 | 7.31.E-02 | 9.19.E-01 | 3.51.E-02 | 4.41.E-01 | 2.58.E-02 | 3.24.E-01 | **9.92.E-01** | **4.76.E-01** | **3.50.E-01** |
| 1.17.E+00 | 2.46.E-03 | 5.60.E-01 | 1.58.E-03 | 4.11.E-01 | 1.40.E-03 | 1.67.E-01 | 1.00.E+00 | 8.02.E-02 | 4.81.E-01 | 5.89.E-02 | 3.53.E-01 | **1.17.E+00** | **5.62.E-01** | **4.12.E-01** |
| 5.42.E-01 | 1.14.E-03 | 2.60.E-01 | 7.32.E-04 | 1.91.E-01 | 6.50.E-04 | 0.00.E+00 | 5.43.E-01 | 0.00.E+00 | 2.61.E-01 | 0.00.E+00 | 1.91.E-01 | **5.43.E-01** | **2.61.E-01** | **1.91.E-01** |
| 1.21.E+00 | 2.56.E-03 | 5.83.E-01 | 1.64.E-03 | 4.27.E-01 | 1.46.E-03 | 1.10.E-01 | 1.11.E+00 | 5.26.E-02 | 5.31.E-01 | 3.86.E-02 | 3.90.E-01 | **1.22.E+00** | **5.84.E-01** | **4.29.E-01** |
| 1.12.E+00 | 2.36.E-03 | 5.38.E-01 | 1.51.E-03 | 3.94.E-01 | 1.34.E-03 | 9.92.E-02 | 1.02.E+00 | 4.76.E-02 | 4.91.E-01 | 3.50.E-02 | 3.61.E-01 | **1.12.E+00** | **5.39.E-01** | **3.96.E-01** |
| 8.59.E-01 | 1.81.E-03 | 4.13.E-01 | 1.16.E-03 | 3.03.E-01 | 1.03.E-03 | 2.98.E-01 | 5.64.E-01 | 1.43.E-01 | 2.71.E-01 | 1.05.E-01 | 1.99.E-01 | **8.61.E-01** | **4.14.E-01** | **3.04.E-01** |
| 7.29.E-01 | 1.54.E-03 | 3.50.E-01 | 9.85.E-04 | 2.57.E-01 | 8.75.E-04 | 8.35.E-02 | 6.47.E-01 | 4.01.E-02 | 3.11.E-01 | 2.94.E-02 | 2.28.E-01 | **7.31.E-01** | **3.51.E-01** | **2.58.E-01** |
| 1.51.E+00 | 3.18.E-03 | 7.23.E-01 | 2.03.E-03 | 5.30.E-01 | 1.81.E-03 | 8.87.E-02 | 1.42.E+00 | 4.26.E-02 | 6.82.E-01 | 3.13.E-02 | 5.00.E-01 | **1.51.E+00** | **7.25.E-01** | **5.32.E-01** |
| 1.00.E+00 | 2.11.E-03 | 4.80.E-01 | 1.35.E-03 | 3.52.E-01 | 1.20.E-03 | 1.46.E-01 | 8.56.E-01 | 7.02.E-02 | 4.11.E-01 | 5.15.E-02 | 3.02.E-01 | **1.00.E+00** | **4.81.E-01** | **3.53.E-01** |
| 1.05.E+00 | 2.21.E-03 | 5.03.E-01 | 1.41.E-03 | 3.69.E-01 | 1.26.E-03 | 5.06.E-01 | 5.43.E-01 | 2.43.E-01 | 2.61.E-01 | 1.78.E-01 | 1.91.E-01 | **1.05.E+00** | **5.04.E-01** | **3.70.E-01** |
| 2.57.E+00 | 5.42.E-03 | 1.23.E+00 | 3.47.E-03 | 9.04.E-01 | 3.08.E-03 | 6.79.E-02 | 2.51.E+00 | 3.26.E-02 | 1.20.E+00 | 2.39.E-02 | 8.83.E-01 | **2.57.E+00** | **1.24.E+00** | **9.07.E-01** |
| 3.56.E+00 | 7.51.E-03 | 1.71.E+00 | 4.80.E-03 | 1.25.E+00 | 4.27.E-03 | 2.24.E-01 | 3.34.E+00 | 1.08.E-01 | 1.60.E+00 | 7.91.E-02 | 1.18.E+00 | **3.56.E+00** | **1.71.E+00** | **1.26.E+00** |
| 4.34.E+00 | 9.17.E-03 | 2.09.E+00 | 5.87.E-03 | 1.53.E+00 | 5.21.E-03 | 1.77.E-01 | 4.18.E+00 | 8.52.E-02 | 2.01.E+00 | 6.25.E-02 | 1.47.E+00 | **4.35.E+00** | **2.09.E+00** | **1.53.E+00** |

**List of Supplementary Figures**

**Figure S1**. QQ plots of studied parameters

**Figure S2.** Correlation cofficient matrix of various groundwater quality parameters.

**Figure S3**. Drinking water quality distribution map

|   **a** |
| --- |
|   **b** |

**Figure S4**. Biplot of Ca^2+^/Na^+^ versus (a) HCO_3_^–^/Na^+^ and (b) Mg^2+^/Na^+^ in the study area

|  |  |
| --- | --- |
|  |  |
|  |  |

**Figure S5**. Contribution of chemical species causing noncancerous risk (HI) to the residents.

**Figure S6**. Health risk index to the residents of the study area
